# Age and sex influence antibody profiles associated with tuberculosis progression

**DOI:** 10.1038/s41564-024-01678-x

**Published:** 2024-04-24

**Authors:** Leela R. L. Davies, Chuangqi Wang, Pia Steigler, Kathryn A. Bowman, Stephanie Fischinger, Mark Hatherill, Michelle Fisher, Stanley Kimbung Mbandi, Miguel Rodo, Tom H. M. Ottenhoff, Hazel M. Dockrell, Jayne S. Sutherland, Harriet Mayanja-Kizza, W. Henry Boom, Gerhard Walzl, Stefan H. E. Kaufmann, Elisa Nemes, Thomas J. Scriba, Douglas Lauffenburger, Galit Alter, Sarah M. Fortune

**Affiliations:** 1 Ragon Institute of MGH, MIT, and Harvard, Cambridge, MA USA; 2 Brigham and Women’s Hospital, Boston, MA USA; 3 Department of Immunology and Microbiology, University of Colorado Anschutz Medical Campus, Aurora, CO USA; 4 Biological Engineering, Massachusetts Institute of Technology, Cambridge, MA USA; 5 South African Tuberculosis Vaccine Initiative and Institute of Infectious Disease and Molecular Medicine, Division of Immunology, Department of Pathology, University of Cape Town, Cape Town, South Africa; 6 Wellcome Centre for Infectious Diseases Research in Africa, Institute of Infectious Disease and Molecular Medicine and Division of Immunology, Department of Medicine, University of Cape Town, Cape Town, South Africa; 7 Department of Infectious Diseases, Leiden University Medical Center, Leiden, the Netherlands; 8 Vaccines and Immunity Theme, Medical Research Council Unit The Gambia at the London School of Hygiene and Tropical Medicine, Banjul, The Gambia; 9 Department of Medicine and Department of Microbiology, Makerere University, Kampala, Uganda; 10 Tuberculosis Research Unit, Case Western Reserve University, Cleveland, OH USA; 11 Department of Science and Technology National Research Foundation Centre of Excellence for Biomedical Tuberculosis Research, South African Medical Research Council Centre for Tuberculosis Research, Division of Molecular Biology and Human Genetics, Faculty of Medicine and Health Sciences, Stellenbosch University, Cape Town, South Africa; 12 Max Planck Institute for Infection Biology, Berlin, Germany; 13 Max Planck Institute for Multidisciplinary Sciences, Göttingen, Germany; 14 Hagler Institute for Advanced Study, Texas A&M University, College Station, TX USA; 15 Moderna Therapeutics, Cambridge, MA USA; 16 Department of Immunology and Infectious Diseases, Harvard T.H. Chan School of Public Health, Boston, MA USA

**Keywords:** Antibodies, Tuberculosis

## Abstract

Antibody features vary with tuberculosis (TB) disease state. Whether clinical variables, such as age or sex, influence associations between *Mycobacterium tuberculosis*-specific antibody responses and disease state is not well explored. Here we profiled *Mycobacterium tuberculosis*-specific antibody responses in 140 TB-exposed South African individuals from the Adolescent Cohort Study. We identified distinct response features in individuals progressing to active TB from non-progressing, matched controls. A multivariate antibody score differentially associated with progression (SeroScore) identified progressors up to 2 years before TB diagnosis, earlier than that achieved with the RISK6 transcriptional signature of progression. We validated these antibody response features in the Grand Challenges 6–74 cohort. Both the SeroScore and RISK6 correlated better with risk of TB progression in adolescents compared with adults, and in males compared with females. This suggests that age and sex are important, underappreciated modifiers of antibody responses associated with TB progression.

## Abstract (web summary)

Analysis of *Mycobacterium tuberculosis*-specific antibody responses in previously exposed South African cohorts reveals that profile features associated with progression to active tuberculosis are affected by age and sex.

## Main

Up to a quarter of the world’s population is estimated to have been infected with *Mycobacterium tuberculosis* (*Mtb*), the aetiologic agent of tuberculosis (TB), and one of the deadliest global pathogens^1^. Only 5–10% of individuals with immune sensitization to *Mtb* consistent with infection ever develop the morbidity, mortality and transmission risks associated with active TB^2^. Identifying individuals at risk of progression to active disease is a cornerstone of the World Health Organization’s End TB Strategy^3^. However, the immunology of TB progression remains incompletely understood, and current diagnostics—the tuberculin skin test (TST) and interferon gamma release assay (IGRA)—correlate poorly with progression^4–9^. Better understanding of the range of immune phenotypes of individuals destined to progress to active TB is urgently needed.

Recent proteomic, metabolomic and transcriptomic studies have defined markers of progression to incident TB disease^10–16^. Multi-gene transcriptomic signatures robustly identify individuals at greatest risk of TB progression^12,14,15,17^, collectively pointing to an expanded inflammatory response marked by complement activation and type I and II interferon (IFN) signalling that increases approaching the time of TB disease manifestation^18^. IFN has been implicated in *Mtb* pathogenesis both in mouse models and in human patients with TB^19–21^, and it has been proposed that the identified inflammatory transcriptomic signatures reflect early or subclinical TB disease^14,18^. However, these signatures are not pathogen specific and may be influenced by other inflammatory states^22,23^. A recent study demonstrated the existence of *Mtb-*specific adaptive immune correlates of progression in distinct antigen-specific T cell receptor (TCR) repertoires that accumulate in progressors versus non-progressors^24^.

We and others have demonstrated that *Mtb*-specific antibody signatures vary across TB clinical states^25–33^. These studies have revealed unrecognized immune phenotypes, including the presence of *Mtb*-specific humoral responses in the so-called resisters, who are highly exposed to *Mtb* but do not mount the sustained IFN-dominated T cell response that underlies the diagnostic TST and IGRA tests^34,35^. The effects of human immunodeficiency virus (HIV) on humoral immune responses have been assessed^25,30^. However, the impact of other clinical variables such as age and sex on antibody responses in *Mtb*-infected people, or indeed on other TB immune phenotypes, is largely unexplored.

In this Article, we used a systems serology approach to investigate the association of *Mtb*-specific antibody responses with TB disease progression in a well-characterized longitudinal cohort of HIV-negative, IGRA-positive or TST-positive participants, the Adolescent Cohort Study (ACS)^14^. We identified changes in *Mtb*-specific antibody profiles in progressors compared with non-progressor controls, up to 2 years before the diagnosis of active TB, and assessed the reproducibility of these features in a second cohort, Grand Challenges 6–74 (GC6)^12^. These data indicate that progressors have distinct *Mtb*-specific antibody profiles as compared with non-progressors and that age and sex are critical modifiers of the immune phenotypes that define TB progression.

## Results

### Progressors have distinct *Mtb*-specific antibody responses

**Fig. 1:**
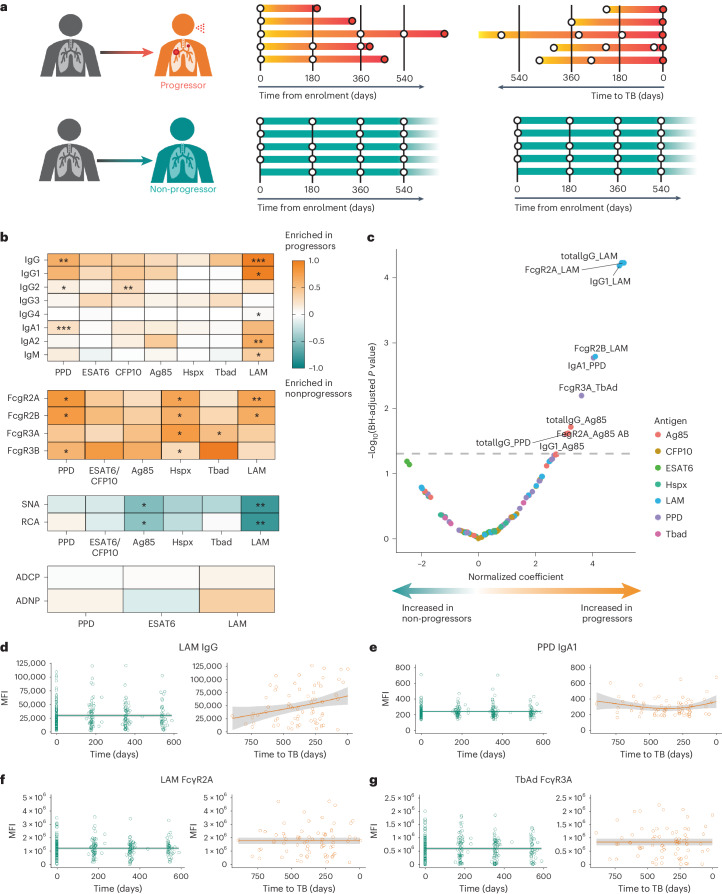
ACS progressors exhibit distinct *Mtb*-specific antibody profiles. **a**, Serum collected longitudinally from a cohort of South African adolescents who later progressed to active TB disease (*n* = 36) or who maintained asymptomatic infection (*n* = 104). For analyses in the current study, progressors were aligned by time of diagnosis and non-progressors by time of enrolment. **b**, Systems serologic assays performed against a panel of *Mtb* antigens, including the selection of antibody isotype and subclasses, the binding of Fcγ receptors, the binding to Fc of lectins SNA (recognizes sialic acid) and RCA (recognizes galactose), and the ability to recruit antibody-mediated cellular phagocytosis (ADCP) and neutrophil phagocytosis (ADNP). For each indicated assay, values for each individual were averaged over time. Each heatmap represents log­_2_(median value in progressors/median value in non-progressors). The statistical significance of the differences between progressors and non-progressors was measured by two-sided Mann–Whitney test followed by Benjamini–Hochberg (BH) correction for multiple comparisons. *P < 0.05; **P < 0.01. **c**, Mixed-effects linear modelling to evaluate the association between antibody features and progressor status by controlling age, sex, ethnicity, school code and time of sample collection. Likelihood ratio test was used to compare the two paired models, and *P* values were corrected for multiple comparisons by the BH method. The *x* axis indicates the effect size as a normalized coefficient of the variable of progression, and the *y* axis −log_10_ of the adjusted *P* values. The dotted line represents the corrected *P* value of 0.05. **d**, Raw values of LAM-specific IgG measurements for all individuals plotted over time from enrolment (non-progressors, teal) or time to TB (progressors, orange). The solid lines indicate a smooth of median values, using a generalized additive model, and the grey shading indicates one standard deviation. **e**–**g**, Plots for PPD-specific IgA1 (**e**), LAM-specific antibody binding of FcγR2A (**f**) and TbAd-specific antibody binding of FcγR3A (**g**). Source data

Changes in *Mtb*-specific antibody levels, isotype selection and Fc glycosylation are sensitive biomarkers of TB disease states^25–29,34,35^. We therefore sought to determine the extent to which *Mtb*-specific antibody profiles, which can distinguish active TB from latent infection^26,30^, pre-date the diagnosis of active TB. We comprehensively profiled the *Mtb*-specific humoral immune response in longitudinal serum samples collected from 36 adolescent progressors before the diagnosis of active TB and 104 matched non-progressors from the ACS cohort^14^ (Fig. 1a and Supplementary Table 1). Averaged across all timepoints, overall levels of *Mtb*-specific IgG, the dominant isotype in the blood, trended higher in progressors compared with non-progressors (Fig. 1b). Lipoarabinomannan (LAM)-specific IgG, IgG1, IgG4, IgA2 and IgM were all significantly increased in progressors compared with non-progressors, as were purified protein derivative (PPD)-specific IgG, IgG2 and IgA1, and culture filtrate protein 10 (CFP10)-specific IgG2. *Mtb*-specific Fc receptor binding antibodies were also increased in progressors, including LAM-specific FcγR2A and FcγR2B, PPD-specific FcγR2A, FcγR2B and FcγR3B, heat shock protein X (HspX)-specific FcγR2A, FcγR2B, FcγR3A and FcγR3B, and 1-tuberculosinyl adenosine 1 (TbAd)-specific FcγR3A. LAM- and antigen 85 complex (Ag85)-specific Fc binding of lectins *Sambucus nigra* agglutinin (SNA) and *Ricinus communis* agglutinin I (RCA) were selectively increased in non-progressors, indicating elevated Fc sialylation and galactosylation; decreased Fc sialic acid and galactose have been associated with the inflammatory humoral profile of active TB^27^. We assessed antibody-mediated effector functions, but no differences between progressors and non-progressors were identified in antibody-dependent cellular or neutrophil phagocytosis across several *Mtb*-specific antigens.

We next used a mixed linear model to rank and identify the most differential antibody features between progressors and non-progressors, controlling for the effects of demographic confounders and study timepoints (Fig. 1c). The model identified antibody responses targeting LAM as increased in progressors compared with non-progressors, including LAM-specific total IgG, IgG1, FcγR2A and FcγR2B binding levels. PPD-specific total IgG and IgA1 were also selectively enriched in progressors, albeit to a lesser extent, as were Ag85-specific total IgG, IgG1 and FcγR2A, and TbAd-specific FcγR3A binding. By contrast, no measured *Mtb*-specific antibody features were significantly enriched in non-progressors.

### *Mtb*-specific antibody responses vary longitudinally

**Extended Data Fig. 1:**
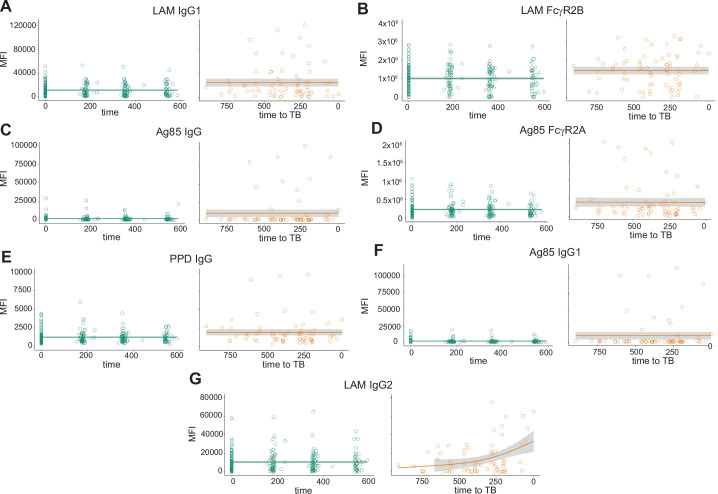
Temporal trajectories of additional Mtb-specific antibody features significantly enriched in ACS progressors. Raw values of measured antibody features for all indivduals were plotted over time from enrollment (non-progressors, teal) or time to TB (progressors, orange). Solid lines indicate a smooth of median values, using a generalized additive model, and grey shading indicates 95% confidence interval. Data is shown for **a**) LAM IgG1, **b**) LAM FcgR2B, **c**) Ag85 IgG, **d**) Ag85 FcgR2A, **e**) PPD IgG, **f**) Ag85 IgG1, and **g**) LAM IgG2. These include all antibody features found to statistically differ between progressors and non-progressors in mixed effects linear modeling, as well as LAM IgG2, which did not statistically differ but is shown for comparison. Source data

Prior analyses of the ACS cohort found that blood transcriptional signatures of progression increased closer to the time of TB diagnosis^12,15,17,18^, whereas the frequency of TCR specificities enriched in progressors remained relatively stable across the study period^24^. We therefore examined the longitudinal evolution of antibody features that were significantly increased in progressors as compared with non-progressors (Fig. 1d–g and Extended Data Fig. 1). The temporal and inter-individual variability of each measured feature was high. In progressors, some antibody features, such as LAM-specific IgG and PPD-specific IgA1, increased at timepoints proximal to TB diagnosis. However, others, such as LAM-specific FcγR2A binding and TbAd-specific FcγR3A binding, remained stably elevated in progressors over the duration of follow-up. Thus, while some measured antibody features increased approaching the time of clinical diagnosis, like the transcriptional signatures, others appeared to be longitudinally stable up to 2 years before TB diagnosis, as observed for TCR specificities.

### An *Mtb*-specific SeroScore detects TB progression risk

**Fig. 2:**
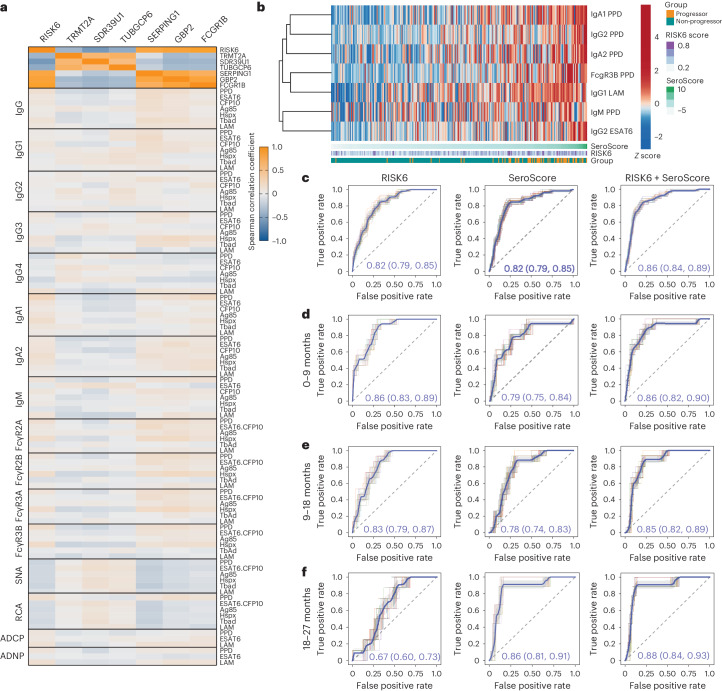
An *Mtb*-specific SeroScore differentiates progressors from non-progressors. **a**, For all individuals at all timepoints, Spearman correlations were calculated between all measured *Mtb*-specific antibody features and RISK6 score (*n* = 377 measurements) or transcript expression levels of each of its six components (*n* = 312 measurements). The heatmap indicates Spearman correlation coefficient for each comparison. **b**, A multivariate SeroScore was developed on the basis of systems serology data in ACS. The heatmap represents *Z*-scored data for the six features included in the SeroScore. Each column represents one individual (*n* = 36 progressors and *n* = 104 non-progressors). Individuals are sorted by overall SeroScore as shown in the track beneath the heatmap. RISK6 score and progressor/non-progressor status of each individual are also indicated in tracks. **c**, ROC curves developed assessing the ability to differentiate progressors (*n* = 29) from non-progressors (*n* = 99) of RISK6 (left), SeroScore (middle) and both RISK6 and SeroScore in combination (right). ROC curves were generated 50 times using randomly selected 80% of samples with group stratification. The mean curve is indicated in blue, with grey shading indicating one standard deviation. The mean AUC with 95% confidence interval is indicated. **d**–**f**, Additional ROC curves developed only including progressors in time windows 0–9 months before diagnosis (*n* = 19 progressors) (**d**), 9–18 months before diagnosis (*n* = 18 progressors) (**e**) and 18–27 months before diagnosis (*n* = 10 progressors) (**f**). Source data

A parsimonious transcriptomic signature was previously developed, RISK6, which differentiates progressors from non-progressors in the ACS cohort^15^. RISK6 signature scores were determined by a pair-ratio approach using three transcripts upregulated in progressors (SERPING1, GBP2 and FCGR1B) and three downregulated in progressors (TRMT2A, SDR39U1 and TUBGCP6). We next asked whether the measured differential antibody features captured the same or distinct immunological processes as those marked by RISK6, by measuring correlations between *Mtb*-specific antibody features and RISK6 scores and transcript expression levels. The two sets of RISK6 transcripts showed the expected correlations with each other, consistent with how they were identified (Fig. 2a). However, the measured antibody features showed minimal correlation with RISK6 and its individual component transcripts (Fig. 2a), suggesting that *Mtb*-specific antibody profiles capture distinct biologic processes.

**Extended Data Fig. 2:**
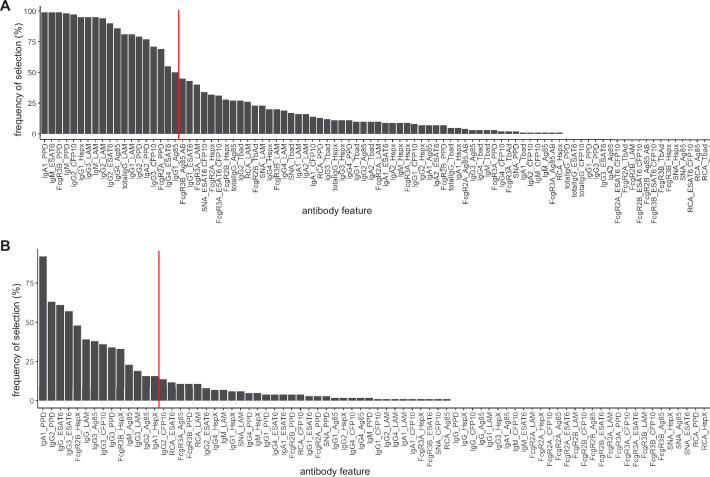
Selection of antibody features for inclusion in SeroScores. For **a**) ACS, and **b**) GC6, LASSO (Least Absolute Shrinkage and Selection Operator) regularization was applied to 100 randomly selected subsets, each containing 80% of the full dataset, and iterated 10 times. The frequency of selection of each antibody feature is shown. Red lines indicate the threshold defining the features that were evaluated in combination for each SeroScore. Source data

We next defined a minimal set of antibody features associated with progression. We used the least absolute shrinkage and selection operator (LASSO) technique to identify the most relevant features differentiating progressor and non-progressor groups and identified the combination of features with the highest discriminative ability (Extended Data Fig. 2a). This analysis generated a minimal set of seven features (PPD-specific IgG2, IgA1, IgA2, IgM, FcγR3B, LAM-specific IgG1 and early secretory antigen 6 (ESAT6)-specific IgG2) that enabled resolution of progressors and non-progressors in ACS (Fig. 2b). We used these seven features to define a SeroScore, a multivariate score differentially associated with progression.

To further understand whether the SeroScore and RISK6 captured similar or different biologic processes, we compared their ability to differentiate progressors and non-progressors over time. Across the full study period, RISK6 and SeroScore similarly differentiated progressors, each with an area under the curve (AUC) of 0.82 independently, which improved to 0.86 in combination (Fig. 2c). At 0–9 months, the time window most proximal to diagnosis of active TB, the SeroScore had a median AUC of 0.79, compared with 0.86 for RISK6; combining the two scores in this timeframe did not improve performance beyond that of RISK6 alone (Fig. 2d). At 9–18 months (Fig. 2e) and 18–27 months (Fig. 2f), RISK6 performance declined to AUC of 0.83 and 0.67, respectively, but the SeroScore remained stable with AUC of 0.78 and 0.86. These findings suggest that, particularly at timepoints more remote from diagnosis, the SeroScore captured biologic information that RISK6 did not.

### Sex influences scores associated with progression

**Fig. 3:**
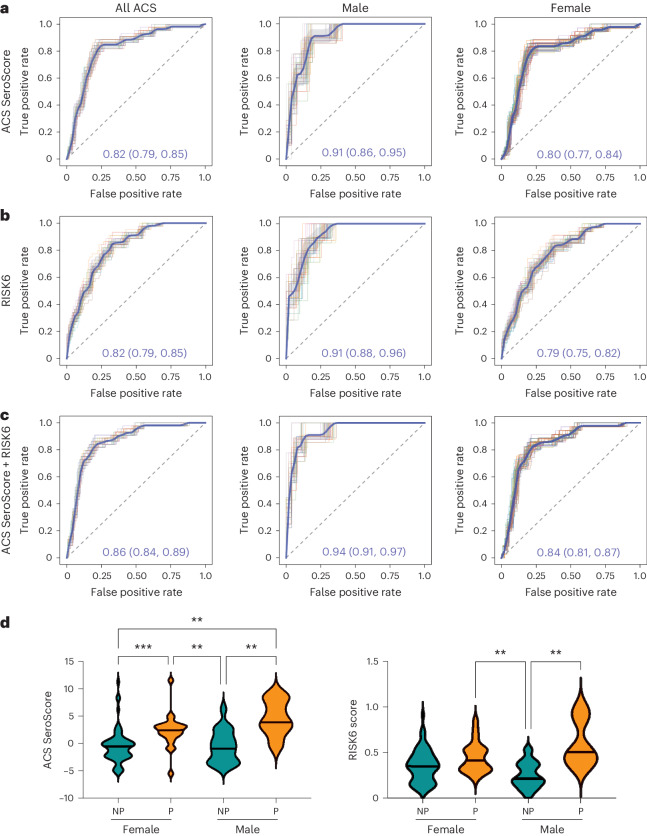
Sex modulates the association of SeroScore and RISK6 with TB progression. ROC curves were developed measuring the ability of the ACS-derived SeroScore and RISK6 to differentiate progressors from non-progressors in ACS. **a**, The identification of progressors by the ACS-derived SeroScore among all ACS individuals (*n* = 29 progressors and *n* = 99 non-progressors), males only (*n* = 7 progressors and *n* = 37 non-progressors) and females only (*n* = 22 progressors and *n* = 63 non-progressors). **b**, As in **a**, the performance of RISK6 among all ACS, males only and females only. **c**, The identification of progressors by the ACS-derived SeroScore and RISK6 in combination among all ACS, males only and females only. For **a**–**c**, the mean of 50 curves is shown in blue, with grey shading indicating one standard deviation. The mean AUC with 95% confidence interval is indicated on each plot. **d**, The ACS SeroScore and RISK6 signature score were plotted for female (*n* = 22) and male (*n* = 7) progressors (P, orange) and female (*n* = 63) and male (*n* = 37) non-progressors (NP, teal) from ACS. The groups were compared by Kruskal–Wallis test, with *P* values <0.05 indicated. Source data

Sex is a well-established modifier of immune responses^36–38^, and the global prevalence of active TB in males exceeds that in females with a ratio of 1.7 (ref. ^39^). We sought to determine whether sex influenced the association of the SeroScore or the RISK6 signature with progression in the ACS cohort, which is 67.1% female. Across the full study period, both the SeroScore and RISK6 identified male progressors slightly better than females (Fig. 3a,b). We then combined the SeroScore and RISK6 linearly to determine whether they had better ability to identify progressors in combination. The combined score was better able to identify progressors in both male and female subgroups, but the mean AUC among female participants, at 0.84, remained lower than among male participants, where it reached 0.94 (Fig. 3c). When we directly plotted SeroScore by sex and progressor status, male progressors trended towards higher SeroScores and RISK6 scores than female progressors, while both scores were similar in male and female non-progressors (Fig. 3d). Thus, despite being discovered in a predominantly female cohort, both the SeroScore and RISK6 signatures captured progression better among males than females.

### The ACS SeroScore detects TB progression among adolescents

**Extended Data Fig. 3:**
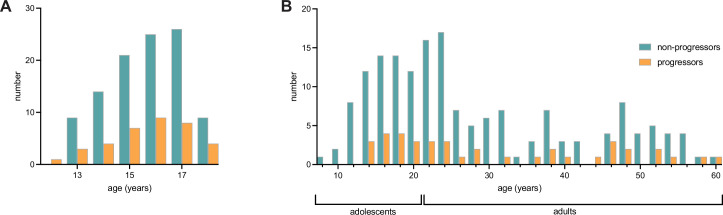
Age distributions for included subjects from ACS and GC6. **a**) Histogram of age distributions for ACS progressors and non-progressors. **b**) Histogram of age distributions for GC6 progressors and non-progressors. For GC6 subjects, adolescents were defined as those with age at enrollment between 8 and 20 years, and adults were those with age at enrollment of 21 or more years. Source data

We next sought to assess the performance of the ACS SeroScore in a second cohort of 39 progressors and 169 non-progressors from the South African subcohort of GC6, a longitudinal study of TB household contacts^12^. Prior work demonstrated that the ACS-defined RISK6 signature had reduced performance in the GC6 cohort, which was attributed to differences in study design, environmental or temporal exposure to TB, geography of the participants and wider age range (Supplementary Table 2 and Extended Data Fig. 3)^15^.

**Fig. 4:**
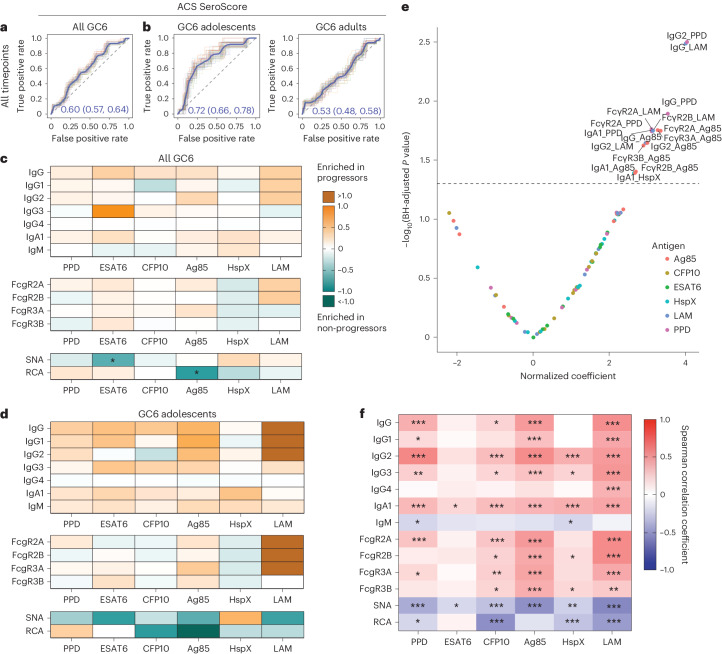
*Mtb*-specific antibody profiles correlate with progression in GC6 adolescents. **a**, ROC curves developed to evaluate the ability of the ACS-derived SeroScore to differentiate progressors (*n* = 39) and non-progressors (*n* = 169) among individuals from the GC6 cohort. The grey shading indicates one standard deviation. The mean AUC is indicated. **b**, ROC curves evaluating the ability of the ACS-derived SeroScore to differentiate progressors from non-progressors among GC6 adolescents (age 8–20 years at enrolment, *n* = 14 progressors and *n* = 63 non-progressors) and adults (age >20 years at enrolment, *n* = 25 progressors and *n* = 106 non-progressors). The mean of 50 curves is shown in blue, with grey shading indicating one standard deviation. The mean AUC with 95% confidence interval is indicated. **c**, *Mtb*-specific systems serology used to profile serum collected longitudinally from the GC6 cohort. For each indicated assay, values for each individual were averaged over time. In the heatmap, each cell represents log_2_(median value in progressors/median value in non-progressors). The statistical significance of the differences between progressors and non-progressors was measured by two-sided Mann–Whitney test followed by Benjamini–Hochberg (BH) correction for multiple comparisons. *P < 0.05 after correction. **d**, A heatmap representing averaged values over time for adolescent individuals only. Each cell represents log_2_(median value in progressors/median value in non-progressors). The statistical significance of the differences between progressors and non-progressors were measured by two-sided Mann–Whitney test followed by BH correction for multiple comparisons. *P < 0.05 after correction. **e**, Among all GC6 individuals, mixed-effects linear modelling was used to evaluate the association between antibody features and progressor status by controlling age, sex and time of sample collection. Likelihood ratio test was used to compare the two paired models, and *P* values were corrected for multiple comparisons by the BH method. The *x* axis indicates effect size as normalized coefficient of the variable of progression, and the *y* axis −log_10_ of the adjusted *P* values. The dotted line represents the corrected *P* value of 0.05. **f**, A heatmap representing Spearman correlation coefficients between each antibody feature and age among GC6 non-progressors only (*n* = 169). *P* values for each correlation were adjusted for multiple comparisons by the BH method, and adjusted *P* values are indicated: *P < 0.05, **P < 0.01, ***P < 0.001. Source data

**Extended Data Fig. 4:**
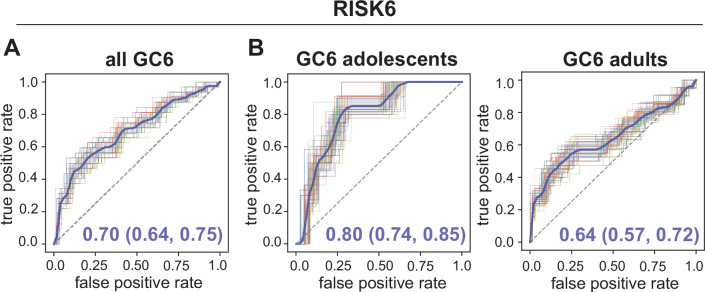
Ability of RISK6 to identify GC6 adolescent and adult progressors. ROC curves were developed over the full study duration for the subset of individuals in GC6 for whom the RISK6 score was available. A) ROC curve indicates performance of RISK6 among all GC6 individuals (n = 35 progressors and n = 135 non-progressors). B) ROC curve for performance of RISK6 among GC6 adolescents (age 8–20 years, n = 12 progressors and n = 49 non-progressors) and adults (age ≥21 years, n = 23 progressors and n = 86 non-progressors). ROC curves were generated 50 times using randomly selected 80% of samples with group stratification. The mean curve is indicated in blue, with grey shading indicating one standard deviation. Mean AUC with 95% confidence interval is indicated. Source data

**Extended Data Fig. 5:**
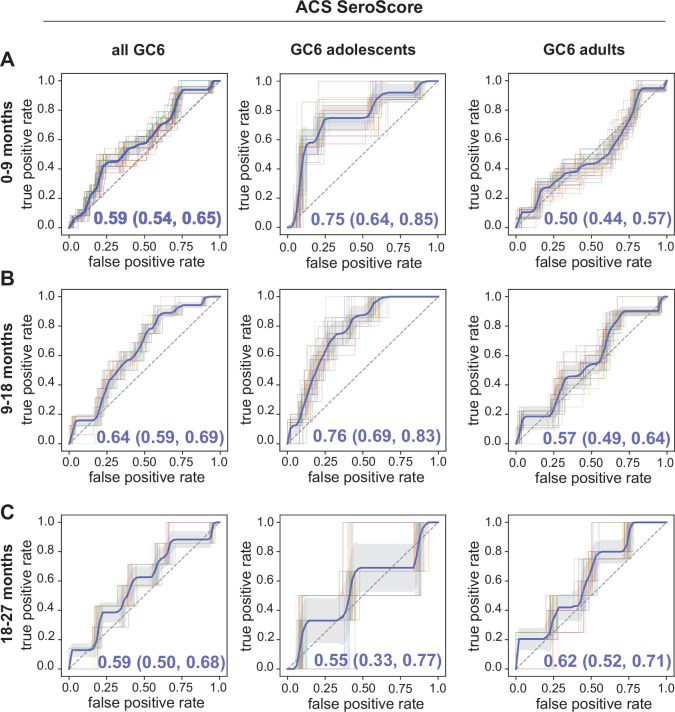
Longitudinal ability of the ACS-derived SeroScore to identify GC6 adolescent and adult progressors. The SeroScore derived in ACS was used to develop ROC curves for GC6 adolescents (age 8–20 years) and adults (≥21 years) over time windows **a**) 0–9 months, **b**) 9–18 months, and **c**) 18–27 months prior to diagnosis of active TB. ROC curves were generated 50 times using randomly selected 80% of samples with group stratification. The mean curve is indicated in blue, with grey shading indicating one standard deviation. Mean AUC with 95% confidence interval is indicated. Source data

When applied to the GC6 cohort, the ACS-derived SeroScore identified progressors marginally, with an overall mean AUC of 0.60 (Fig. 4a). To more closely align the demographic features of GC6 with ACS, we stratified the GC6 subjects into adolescents, who were 8–20 years of age at enrolment (14 progressors and 63 non-progressors), and adults, who were 21–60 years of age at enrolment (25 progressors and 106 non-progressors). The AUC of the SeroScore differed with age, with a mean value of 0.72 among adolescents and only 0.53 among adults (Fig. 4b). Interestingly, among individuals with an available RISK6 score, the RISK6 test performance also improved among the adolescent GC6 group (Extended Data Fig. 4). As in the ACS cohort, the ability of the ACS-derived SeroScore to identify progressors remained longitudinally stable in GC6 up to 18 months before diagnosis, but the small sample size limited evaluation at timepoints earlier than 18 months (Extended Data Fig. 5).

Given the increased heterogeneity of the GC6 cohort, we next aimed to independently determine whether distinct *Mtb*-specific antibody features associated with progression existed in GC6. Humoral differences between progressors and non-progressors were more subtle in the GC6 cohort compared with ACS (Fig. 4c). Among adolescents, several *Mtb*-specific antibody responses were selectively enriched among progressors, including markedly increased LAM-specific IgG1, IgG2 and IgG3 levels and binding of FcγR2A, FcγR2B and FcγR3A, though, given the small sample size, none achieved statistical significance after multiple test correction. As observed in ACS, Fc sialylation and galactosylation trended towards an enrichment in non-progressors (Fig. 4d). In a mixed linear model, IgG and IgA1 responses to PPD, LAM and Ag85, and FcγR binding to these antigen-specific antibodies, were significantly enriched in progressors (Fig. 4e). Thus, while the differences were smaller in magnitude, the specific discriminatory *Mtb*-specific antibody profiles observed across progressors and non-progressors in GC6 resembled features identified in the ACS cohort (Fig. 1).

### Class-switched *Mtb* antibodies emerge with increasing age

We postulated that the reduced association of antibody features with progression among GC6 adults might be due to chronic *Mtb* exposure increasing background *Mtb*-specific antibody levels among non-progressors. We measured the relationship between antibody features and the age at the time of enrolment in the non-progressors in GC6 (Fig. 4f). We found that class-switched antibody responses, primarily IgG2, IgG3 and IgA1, positively correlated with age at enrolment. Conversely, IgM levels and Fc sialylation and galactosylation, represented by SNA and RCA, demonstrated negative correlations with age. Together, these data demonstrate that, with increasing age, individuals without known active TB disease exhibit broad class-switching of *Mtb*-specific antibody responses and decreased Fc sialylation and galactosylation, antibody features suggestive of increased inflammatory tone^40^.

### The GC6 SeroScore detects progressors across cohorts

**Fig. 5:**
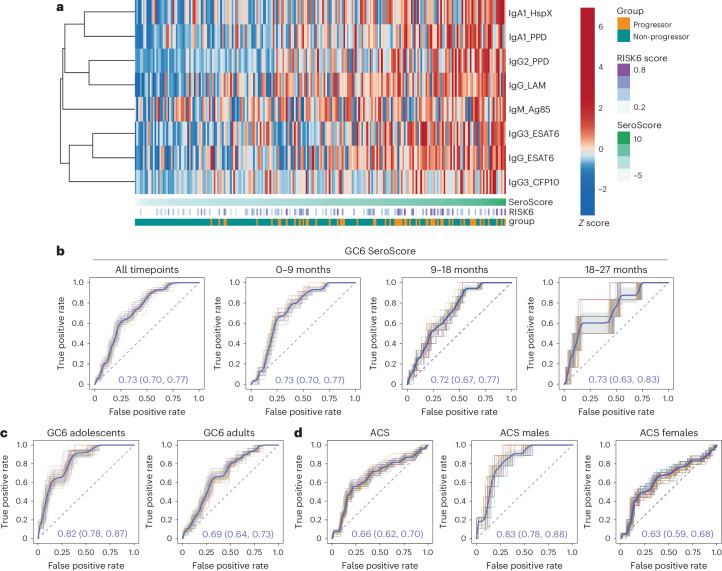
A GC6-derived SeroScore detects humoral correlates of progression. **a**, A multivariate SeroScore was developed in GC6. The heatmap represents *Z*-scored data for the six features included in the SeroScore. Each column represents one individual (*n* = 39 progressors and *n* = 169 non-progressors). The individuals are sorted by overall SeroScore as shown in the track beneath the heatmap. The RISK6 score and progressor/non-progressor status of each individual are also indicated in tracks. **b**, To evaluate the ability of the GC6-derived SeroScore to identify progressors in the same cohort, ROC curves were developed over the total study period (*n* = 39 progressors, *n* = 169 non-progressors) and for progressors in time windows 0–9 months (*n* = 30 progressors), 9–18 months (*n* = 19 progressors) and 18–27 months (*n* = 8 progressors) before the diagnosis of active TB. The mean of 50 curves is shown in blue, with grey shading indicating one standard deviation. The mean AUC with 95% confidence interval is indicated. **c**, ROC curves measure the ability of the GC6-derived SeroScore to identify GC6 progressors in an age-stratified analysis of adolescents (*n* = 14 progressors and *n* = 63 non-progressors) and adults (*n* = 25 progressors and *n* = 106 non-progressors). **d**, The ROC curves measure the ability of the GC6-derived SeroScore to identify progressors in the full ACS cohort (*n* = 29 progressors and *n* = 99 non-progressors), males only (*n* = 7 progressors and *n* = 37 non-progressors) and females only (*n* = 22 progressors and *n* = 63 non-progressors). Source data

Finally, we hypothesized that profiles of progression identified in a more heterogeneous population would be more likely to yield an epidemiologically concordant score of TB progression. We therefore defined an independent SeroScore in GC6 (Extended Data Fig. 2b) with markers that were disproportionately enriched among progressors (Fig. 5a). The LASSO algorithm is designed to avoid selecting multiple co-correlated variables to limit the risk of model overfitting, and thus may select different features for different datasets, even when the underlying architecture is very similar. Nevertheless, the GC6-derived SeroScore included some of the same features as the ACS-derived SeroScore, including PPD-specific IgA1 and IgG2. Similarly, LAM- and ESAT6-specific total IgG were included in the GC6 SeroScore, whereas subclasses LAM-specific IgG1 and ESAT6-specific IgG2 had been included in the ACS SeroScore.

The GC6-derived SeroScore differentiated progressors from non-progressors in GC6 at all tested time windows, with a longitudinally stable mean AUC of 0.72–0.73 at 0–9 months, 9–18 months and 18–27 months, and over all time windows (Fig. 5b). Similar to the ACS SeroScore, the GC6-derived SeroScore identified progressors better among adolescents than adults, with mean AUC values of 0.82 and 0.69, respectively (Fig. 5c). The G6C SeroScore was able to discriminate progressors in ACS, with an AUC of 0.66 overall (Fig. 5d). It also performed better in males, with an AUC of 0.83 as compared with females with an AUC of 0.63. These findings highlight the presence of similar humoral features differentiating progressors from non-progressors in both the ACS and GC6 cohorts, including enhanced expansion of class-switched *Mtb-*specific IgA and IgG2, and higher levels of LAM-specific antibodies in progressors. We also find that age and sex modify the relationship between these *Mtb*-specific humoral features and TB progression.

## Discussion

An explosion of novel profiling tools has begun to define correlates of progression in individuals who go on to develop TB disease^12–18,24^. Here, we investigated the association of *Mtb*-specific humoral profiles with TB progression in a well-characterized cohort of HIV-negative, IGRA-positive South African adolescents^14^. A multivariate *Mtb*-specific SeroScore associated with progression was longitudinally stable for the study duration, up to 2 years before TB diagnosis. The SeroScore was marked by elevated levels of *Mtb-*specific antibodies including class-switched IgG and IgA isotypes, Fc receptor-binding antibodies, and reduced Fc sialylation and galactosylation. The ACS SeroScore was also associated with risk of progression in the adolescent participants from a more epidemiologically diverse cohort of household contacts, GC6, but it poorly identified progression among adults. In addition, both the ACS SeroScore and a SeroScore derived from GC6 participants better differentiated male as compared with female progressors. Surprisingly, we also found that the performance of the well-studied RISK6 transcriptional signature was modulated by age and sex. Thus, age and sex are critical modifiers of these antibody responses and other immune phenotypes associated with TB progression.

The ability of both SeroScores and RISK6 to detect progressors better among adolescents that adults suggests that adolescent progressors harbour unique immune phenotypes. The observed age-associated increases in class-switched IgA and IgG to multiple antigens, including the relatively *Mtb*-specific antigen CFP10, suggest that adults have experienced prior exposures to *Mtb*. Alternatively, the observed differences may reflect adolescents’ relatively more recent *Mtb* exposure^41,42^ or age-specific differences in B and T helper cell responses^43^.

Similarly, both transcriptomic and serologic scores were less able to identify progressors among females. Sex has been linked to extensive differences in both innate and adaptive immunity, including altered complement activity, higher IFN levels and increased antibody responses in females^36–38^ that may affect transcriptomic and serologic signatures. Additional epidemiologic factors that may affect *Mtb*-specific humoral responses, such as co-morbidities including HIV co-infection, geography and race^44–46^, were not evaluated here. While the focus of many clinical correlate studies is the similarity of biomarkers across cohorts, understanding how immune correlates vary across epidemiologically distinct groups may elucidate population-specific determinants of disease risk.

The longitudinal stability of several of the observed serologic responses in progressors is reminiscent of the longitudinal stability of the TCR specificities elevated in the ACS progressors^24^. These longstanding adaptive immune phenotypes suggest that progressor status is determined very early on. It is possible that these responses reflect a higher or more persistent lung mycobacterial burden in progressors before development of symptoms; however, it is also possible that they mark early qualitative differences between a protective and non-protective immune response. Other antibody features, including LAM-specific IgG2 and PPD-specific IgA1, mirror the increase of inflammatory transcriptomic signatures towards the onset symptomatic TB^13–15,18^, suggesting they may be more sensitive markers of B cell surveillance of increasingly inflamed lung tissue. Future longitudinal studies will be essential in detailing the temporal interplay of *Mtb* culture positivity, lung pathology and symptom onset with signatures of progression.

Although antibody correlates of progression have not previously been examined, prior studies of individuals with active TB have identified heterogeneous, broadly increased *Mtb*-specific IgG levels compared with asymptomatic controls, correlating with their bacterial burden^47–50^. Systems serology approaches have shown that active TB is defined by increased levels of IgG across many *Mtb* antigens, higher levels of FcR binding and more inflammatory, less sialylated and galactosylated Fc glycoforms^25–27,30,40^. The antibody features differentially associated with progressors here resemble those differentially enriched in active TB. It is possible that the emergence of these antibody features in progressors long before the diagnosis of active TB reflects smouldering, tissue-level disease. It remains unclear whether the differences in *Mtb*-specific antibodies that distinguish progressors and non-progressors play functional roles in control of infection, and this is an active area of ongoing investigation.

In sum, we have shown here that distinct antibody features are associated with TB disease progression in South African adolescents in an age- and sex-dependent fashion. We find evidence for early *Mtb*-specific humoral immune responses that may mark longstanding subclinical disease, and other antibody features that increase closer to the time of TB diagnosis. The observed alterations in humoral responses among progressors provide critical insights into the immunology of TB progression and emphasize the importance of clinical variables in modulating immune phenotypes of TB disease states.

## Methods

### Study design

#### ACS

The ACS was a prospective cohort study that enrolled 6,363 healthy, HIV-negative South African adolescents aged 12–18 years^14^. Among participants with evidence of *Mtb* infection, diagnosed by a positive QuantiFERON TB Gold in-tube assay or positive TST, progressors were those who developed active intrathoracic TB during the follow-up period, defined by either two sputum smears positive for acid-fast bacilli or one positive sputum culture with microbiologically confirmed *Mtb*. For each progressor, at least two QuantiFERON-positive non-progressors were matched by age at enrolment, sex, ethnic origin, school of attendance and presence or absence of previous episodes of TB disease. Adolescents provided written, informed assent, and parents or legal guardians provided written, informed consent. The original clinical study was reviewed and approved by the Human Research Ethics Committee of the University of Cape Town, and the systems serology analysis by Massachusetts General Hospital.

All samples from progressors with at least 200 ml of available serum were included in the current study (72 samples from 36 individuals). In the absence of prior data on serologic responses in TB disease progression, a formal power calculation was not performed. Sample size was dictated by the number of available progressors in the original cohort and was similar to prior systems serology studies in the setting of HIV or different TB disease states^25,26,30^. At least two non-progressors were matched to each progressor individual by age, sex, school code and ethnicity (264 samples from 104 individuals). Demographics of the included individuals from ACS are presented in Supplementary Table 1.

#### GC6

Samples were additionally included from participants in the GC6 (refs. ^12,16^). Briefly, HIV-negative people aged 10–60 years who had household exposure to an adult with sputum smear-positive TB were enrolled to this study and followed for 2 years. In total, 85.7% of South African subjects were TST positive at baseline^12^. Progressors had intrathoracic TB, defined by sputum culture, smear microscopy and clinical signs. For each progressor, four controls were matched according to recruitment region, age category (≤18 years, 19–25 years, 26–35 years or ≥36 years), sex and year of enrolment. Study protocols were approved by the relevant human research ethics committees. Written informed consent was obtained from participants. For adolescents, consent was obtained from parents or legal guardians of adolescents and written informed assent from each adolescent.

Samples from progressors (114 samples from 39 individuals) and matched non-progressors (458 samples from 169 individuals) were included in the current study. For age-stratified analyses, adolescents were defined as individuals aged 8–20 years (14 progressors and 63 non-progressors), and adults as those older than 20 years (25 progressors and 106 non-progressors). While the original study included South Africa, Gambia, Ethiopia and Uganda, to better control for exposure to similar *Mtb* strains as well as environmental non-tuberculous mycobacteria exposure, only South African participants were included in the current study. Demographics of all included individuals from GC6 are presented in Supplementary Table 2.

### Measurement of biophysical properties of *Mtb*-specific serum antibodies

A customized, multiplex Luminex assay was used to measure *Mtb* antigen-specific antibody responses across multiple isotypes and subclasses. Antigens included commercially available *Mtb* products: PPD (Staten Serum Institute), LAM (BEI Resources NR-14848), ESAT6 (BEI Resources NR-49424), CFP10 (BEI Resources NR-49425), Ag85 (BEI Resources NR-14855), HspX (BEI Resources NR-49428) and TbAd (a generous gift from the laboratory of Dr Branch Moody). An equal mixture of influenza antigens from HA1(B/Brisbane/60/2008) and HA1(H1N1)(A/New Caledonia/20/99) (Immune Technology Corp.) was used as a positive control, and recombinant HA-tagged ebolavirus glycoprotein minus the transmembrane domain (EBOV GPdTM, Mayflower Bioscience 0501-001) was used as a negative control. All peptide antigens were coupled to carboxylate-modified microspheres (Luminex Corp.) by covalent *N*-hydroxysuccinimide (NHS)-ester linkages by 1-ethyl-3-(3-dimethylaminopropyl)carbodiimide hydrochloride (Thermo Fisher Scientific) and Sulfo-NHS (Thermo Fisher Scientific) per the manufacturer’s instructions. Glycan antigens (LAM and TbAd) were first modified in 4-(4,6-dimethoxy-1,3,5-triazin-2-yl)-4-methylmorpholinium chloride (Sigma-Aldrich) at 9.25 mg ml^−1^ at room temperature for 1 h, desalted with a PD-10 column and then incubated with Luminex beads with rotation overnight at room temperature.

Assays were optimized over a dilution curve, to ensure selection of a dilution within the linear range of the assays. A 1:200 dilution was selected to maximize the dynamic range across control samples and to capture the AUC for the full range of dilutions tested. Diluted serum samples were incubated with pooled microspheres for 2 h at room temperature, and then washed three times with phosphate-buffered saline (PBS) with 0.1% bovine serum albumin and 0.05% Tween to wash away unbound antibodies. Secondary detection reagents included phycoerythrin-conjugated goat anti-human IgG, IgG1, IgG2, IgG3, IgG4, IgM, IgA1 and IgA2 (Southern Biotech) and fluorescein-conjugated SNA and RCA (VectorLabs). For FcR binding, recombinant human FcγR2A, Fcγ2B, Fcγ3A and Fcγ3B (Duke University Protein Production Core) were biotinylated using BirA (Avidity) and conjugated to streptavidin-PE (Phycolink). All secondary incubations were performed over 1 h at room temperature. The median fluorescence intensity for each bead region was measured using an iQue Plus Screener (Intellicyt). All samples were assayed in duplicate, and values were averaged. SNA and RCA measurements were normalized to the corresponding IgG measurements.

### Antibody-dependent cellular phagocytosis

Cellular phagocytosis of fluorescent beads coated with PPD, LAM and ESAT6 was performed^51^. The human cell line THP-1 was used to source monocytes from the assay in a reproducible and high-throughput format. Briefly, antigens were biotinylated with 50-fold excess biotin with EZ-link NHS-long chain biotin (Thermo Fisher) following the manufacturer’s instructions, and then adsorbed onto 1 μm fluorescent neutravidin beads (Invitrogen) at a 1:1 (μg:μl) ratio of biotinylated polysaccharide to beads. Ten microlitres of a 1:100 suspension of antigen-coupled beads were added to each well of a 96-well plate along with equal volume of serum diluted 1:30, and plates were incubated for 2 h at 37 °C, and then washed with PBS. A total of 25,000 THP-1 cells (human acute monocytic leukaemia cell line, American Type Culture Collection) were added and incubated at 37 °C for 18–20 h. Cells were fixed with 4% paraformaldehyde before data acquisition. Phagocytosis was measured by iQue Plus Screener (Intellicyt). Phagocytic scores were calculated as (per cent bead-positive cells) × (geometric mean fluorescence intensity (MFI))/10,000. Each sample was assayed in two independent technical replicates and averaged.

### Antibody-dependent neutrophil phagocytosis

Neutrophil phagocytosis was evaluated^52^. To optimize the signal-to-noise ratios, phagocytosis was performed using total donor leukocytes, and analysis was performed on the neutrophil subset. Briefly, as described for cellular phagocytosis, PPD, LAM and ESAT6 were biotinylated and coupled to 1 μm fluorescent neutravidin beads. Ten microlitres of a 1:160 dilution of coupled beads in PBS were opsonized with 10 µl of serum diluted 1:30 at 37 °C for 2 h. Whole blood was collected from healthy donors, red blood cells were lysed with ACK lysis buffer (Quality Biological), and primary leukocytes were isolated by centrifugation and washed in PBS. A total of 50,000 isolated leukocytes were added per well and incubated for 1 h at 37 °C. The cells were then stained with 10 mg ml^−1^ Pacific Blue anti-human CD66b antibody (BioLegend) and fixed in 4% paraformaldehyde before measurement and analysis on the iQue Plus Screener (Intellicyt). Neutrophils were then gated on CD66b^+^, and phagocytic scores were calculated as above. Two healthy leukocyte donors were used as biological replicates for each sample and assayed in parallel, and replicates were averaged.

### Statistics

Univariate comparisons of individual assayed antibody features were performed in GraphPad Prism 9. Progressor and non-progressor groups were compared using two-sided Mann–Whitney tests, followed by multiple test correction with the Benjamini–Hochberg method. Male and female progressor and non-progressor groups were compared using two-sided Kruskal–Wallis tests. All remaining data visualizations and analyses were performed in Python version 3.9.16 or R version 4.0.2.

### Nested mixed linear model

To evaluate the difference of each individual measurement between the progressor and non-progressor groups by controlling the effects of the potential cofounders including demographic features of age, sex and school (ACS only) and the timepoint of sample collection, we used a nested mixed linear model. In detail, we applied two nested mixed linear models (null and full model) without/with progressor group information to assess the significance of the association between measurements and progressor group while controlling for these potential confounding characteristics. We fit two mixed linear models using the maximum likelihood estimation (MLE) and estimated the improvement in model fit by likelihood ratio test (LRT), which follows a chi-square (λ^2^) distribution to identify the associated measurements.

$${{\mathrm{Null}}\; {\mathrm{model}}}:{m}_{{ij}} \sim 1+{{\mathrm{Sex}}}_{j}+{{\mathrm{Age}}}_{j}+{{\mathrm{District}}}_{j}+{{\mathrm{VisitDate}}}_{j}+\left(1|{\mathrm{ID}}_{j}\right)$$

$$\begin{array}{l}{{\mathrm{Full}}\; {\mathrm{model}}}:{m}_{{ij}} \sim 1+{{\mathrm{Sex}}}_{j}+{{\mathrm{Age}}}_{j}+{{\mathrm{District}}}_{j}+{{\mathrm{group}}}_{i}\\+\,{{\mathrm{VisitDate}}}_{j}+(1{\rm{|}}{\mathrm{ID}}_{j})\end{array}$$

$${{\mathrm{Likelihood}}\; {\mathrm{ratio}}}\,{{\mathrm{test}}}:{{\mathrm{LRT}}}=-2\times \frac{{{\mathrm{MLE}}}\,{{\mathrm{in}}}\,{{\mathrm{full}}}\,{{\mathrm{model}}}}{{{\mathrm{MLE}}}\,{{\mathrm{in}}}\,{{\mathrm{null}}}\,{{\mathrm{model}}}} \sim {{\lambda }}^{2}$$

Here, ‘District’, identified by the ‘SchoolCode’, represents the geographical difference, while ‘ID’ denotes individual participant. The R package lme4 was used to fit the mixed linear model to each measurement and test for differences in measurements, depending on whether each sample belongs to progressor group or non-progressor group. The *P* value from the likelihood ratio test was adjusted by multiple testing correction using the Benjamini–Hochberg procedure, and the *t* value (normalized coefficients) associated with the progressor/non-progressor status, ‘Group’ in the full model, were visualized in a volcano plot using the ggplot function in R package ‘ggplot2’ (version 3.3.5).

### Definition of SeroScores

SeroScores were defined as multivariate antibody signatures differentially associated with progressors. To define SeroScores, measurements from ACS or GC6 were log_2_-transformed to correct the skewness of distribution and then *Z*-scored. Measurements were averaged if more than one sample was collected from the same patient within the given time window.

Next, LASSO regularization^53^ was used to select representative features. In detail, 100 sample sets were generated through random sampling. Each sample set included a randomly selected 80% of all samples with group stratification. For each sample set, LASSO feature selection was performed ten times, and features that occurred at least 80% of the time (that is, at least eight among ten times) were selected. The feature selection process was run on 100 generated datasets in parallel. Selected features were ordered by occurrence and the top *K* (typically 12) features were selected as the final candidates. *K* was manually chosen in different experiments. The procedure was implemented in the select_lasso function in the systemsseRology (version 1.1) package in R.

All additive feature combinations from the selected set of *K* candidates were then evaluated exhaustively. The performance of each combination was evaluated by calculating the mean AUC of receiver operating characteristic (ROC) curves generated using a randomly selected 80% of samples for 50 iterations. Feature combinations with the largest mean AUC value were selected to define the SeroScore.

### Measurement of receiver operating characteristics

To evaluate the performance of SeroScores and RISK6, respectively and in combination, we generated ROC curves in various conditions. For each condition, we estimated the mean AUC from 50 runs, where for each run 80% of the samples with group stratification were randomly selected. The 95% confidence interval of the AUC value was estimated as ± two standard deviations from the mean, under the assumption of Gaussian distribution. ROC curve visualization and AUC calculation were implemented on the basis of the functions roc_curve, auc and RocCurveDisplay in the Python package sklearn.metrics (version 1.2.1). The 95% confidence interval was programmed using the function norm.interval in the Python package scipy.stats (version 1.10.1).

### Reporting summary

Further information on research design is available in the Nature Portfolio Reporting Summary linked to this article.

### Supplementary information

Reporting Summary

Supplementary Tables 1 and 2 Summary of demographic information for included subjects from ACS and GC6-74.

### Source data

Source Data Fig. 1 Statistical source data.

Source Data Fig. 2 Statistical source data.

Source Data Fig. 3 Statistical source data.

Source Data Fig. 4 Statistical source data.

Source Data Fig. 5 Statistical source data.

Source Data Extended Data Fig. 1/Table 1 Statistical source data.

Source Data Extended Data Fig. 2/Table 2 Statistical source data.

Source Data Extended Data Fig. 3/Table 3 Statistical source data.

Source Data Extended Data Fig. 4/Table 4 Statistical source data.

Source Data Extended Data Fig. 5/Table 5 Statistical source data.

## Data Availability

Full systems serology datasets for ACS and GC6 are publically available^54^. RISK6 scores used in this paper were previously calculated for both the ACS cohort^15^ and the GC6 cohort (GEO accession number GSE94438). Source data are provided with this paper.

## Code availability

All the code used for analysis in this paper is deposited on Zenodo at 10.5281/zenodo.10637145 (ref. ^54^) and on Github at https://github.com/ChuangqiWang/TB_Progressor (ref. ^55^).
