# Supplementary material for: Age and sex influence antibody profiles associated with tuberculosis progression
Source: Nat Microbiol. 2024 Apr 24;9(6):1513–25. doi: 10.1038/s41564-024-01678-x (PMC11153143; doi:10.1038/s41564-024-01678-x)
Supplement: Supplementary file 1 — Reporting Summary [file 41564_2024_1678_MOESM1_ESM.pdf]

nature portfolio

Corresponding author(s): Sarah M Fortune
Galit Alter

Last updated by author(s): 2024 02 20

Reporting Summary

Nature Portfolio wishes to improve the reproducibility of the work that we publish. This form provides structure for consistency and transparency in reporting. For further information on Nature Portfolio policies, see our Editorial Policies and the Editorial Policy Checklist.

Statistics

For all statistical analyses, confirm that the following items are present in the figure legend, table legend, main text, or Methods section.

n/a	Confirmed	
☐	☒	The exact sample size (*n*) for each experimental group/condition, given as a discrete number and unit of measurement
☐	☒	A statement on whether measurements were taken from distinct samples or whether the same sample was measured repeatedly
☐	☒	The statistical test(s) used AND whether they are one- or two-sided<br>*Only common tests should be described solely by name; describe more complex techniques in the Methods section.*
☐	☒	A description of all covariates tested
☒	☐	A description of any assumptions or corrections, such as tests of normality and adjustment for multiple comparisons
☐	☒	A full description of the statistical parameters including central tendency (e.g. means) or other basic estimates (e.g. regression coefficient) AND variation (e.g. standard deviation) or associated estimates of uncertainty (e.g. confidence intervals)
☐	☒	For null hypothesis testing, the test statistic (e.g. *F*, *t*, *r*) with confidence intervals, effect sizes, degrees of freedom and *P* value noted<br>*Give P values as exact values whenever suitable.*
☒	☐	For Bayesian analysis, information on the choice of priors and Markov chain Monte Carlo settings
☒	☐	For hierarchical and complex designs, identification of the appropriate level for tests and full reporting of outcomes
☒	☐	Estimates of effect sizes (e.g. Cohen's *d*, Pearson's *r*), indicating how they were calculated

Our web collection on statistics for biologists contains articles on many of the points above.

Software and code

Policy information about availability of computer code

Data collection	All systems serology data generated on the iQue Plus Screener was collected using Forecyt software.
Data analysis	Univariate comparisons of individual assayed antibody features were performed in GraphPad Prism 9. Mixed linear models were created using the R package "lme4" and visualized using "ggplot2" version 3.3.5 in R version 4.0.2. LASSO features selection using the R package "systemsseRology". ROC curves were generated, and AUC calculated, in Python using package "sklearn.metrics" version 1.2.1, and confidence intervals programmed using "scipy.stats" version 1.10.1.<br>The code used for the analyses in this paper is available on Github at https://github.com/ChuangqiWang/TB_Progressor.

For manuscripts utilizing custom algorithms or software that are central to the research but not yet described in published literature, software must be made available to editors and reviewers. We strongly encourage code deposition in a community repository (e.g. GitHub). See the Nature Portfolio guidelines for submitting code & software for further information.

Data

Policy information about availability of data

All manuscripts must include a data availability statement. This statement should provide the following information, where applicable:

- Accession codes, unique identifiers, or web links for publicly available datasets
- A description of any restrictions on data availability
- For clinical datasets or third party data, please ensure that the statement adheres to our policy

RISK6 scores included in analyses within the current manuscript were previously calculated for both the ACS cohort (ref 15) and the GC6 cohort (GEO Accession number: GSE94438). All data generated as part of this manuscript will be available in the main text or the supplementary materials. No custom biological materials were generated during the course of this research. All samples were obtained from the researchers who conducted the original ACS and GC6 observational studies, all of whom can be contacted about sample availability from the respective cohorts.

Research involving human participants, their data, or biological material

Policy information about studies with human participants or human data. See also policy information about sex, gender (identity/presentation), and sexual orientation and race, ethnicity and racism.

Reporting on sex and gender	Both male and female participants, by self-reported sex, were included in both ACS and GC6 studies. Included participants from ACS were 67.1% female, and those from GC6 were 58.7% female. Sex-stratified analyses of the measured Mtb-specific antibody responses are key findings presented in the manuscript, and they are shown in Figures 3 and 5.
Reporting on race, ethnicity, or other socially relevant groupings	NA
Population characteristics	ACS is a cohort of HIV-negative South African adolescents aged 12-18 years. GC6 is a cohort of HIV-negative people aged 10–60 years who had household exposure to an adult with sputum smear-positive TB, and all included participants here were from South Africa. Available demographic data for each cohort is described in Supplementary Tables 1 and 2.
Recruitment	See https://www.ncbi.nlm.nih.gov/pmc/articles/PMC5392204/#SD2
Ethics oversight	All clinical research performed in this study was performed in accordance with the Declaration of Helsinki. The clinical samples analyzed in this manuscript were collected as part of the original ACS and GC6-74 observational studies and are described in detail in the original publication and in the Experimental Design section. Samples were collected at all sites only after written informed consent was given by the patients' legal guardian. Subjects in ACS were compensated an amount of R50 (approximately 7 US dollars) in the form of a non-cash payment such as a voucher at every occasion of a blood draw. Subjects in GC6 were compensated for loss of income and transport costs incurred due to research visits. For the ACS cohort study, protocols were approved by the University of Cape Town Research Ethics Committee, Cape Town, South Africa. For the GC6-74 study, protocols were approved by the institutional review boards of Stellenbosch University, Case Western Reserve University, the Uganda National Council for Science and Technology, and the Joint Gambian Government/MRC Ethics Committee. The systems serology analysis was approved by Massachusetts General Hospital.

Note that full information on the approval of the study protocol must also be provided in the manuscript.

Field-specific reporting

Please select the one below that is the best fit for your research. If you are not sure, read the appropriate sections before making your selection.

☒ Life sciences ☐ Behavioural & social sciences ☐ Ecological, evolutionary & environmental sciences

For a reference copy of the document with all sections, see nature.com/documents/nr-reporting-summary-flat.pdf

Life sciences study design

All studies must disclose on these points even when the disclosure is negative.

Sample size	Sample size was determined based on sample availability at the clinical sites where the ACS and G6-74 studies were conducted.
Data exclusions	Raw data were excluded if they failed to meet quality control measures, specifically if the Luminex bead count was less than 20 for a given data point. Otherwise, no data were excluded.
Replication	All assays were performed in two technical replicates, or two biological replicates where applicable, and strong correlation between replicates was confirmed as part of quality control of raw data. The univariate and multivariate antibody signatures identified as associated with progression in the ACS cohort were validated in a second cohort, GC6.
Randomization	Participant allocation was not random, but rather included matched cases and controls. ACS and GC6 were both conducted as large

Randomization	prospective cohort studies. A number of individuals within each study progressed to active TB within the study period. All available samples from these progressors were included in the current study, as well as non-progressors matched by available demographic features.
Blinding	Investigators were blinded as to progressor/non-progressor status during experimental data collection. We performed a supervised computational analysis, and thus analysis was not performed in a blinded manner.

Reporting for specific materials, systems and methods

We require information from authors about some types of materials, experimental systems and methods used in many studies. Here, indicate whether each material, system or method listed is relevant to your study. If you are not sure if a list item applies to your research, read the appropriate section before selecting a response.

Materials & experimental systems

n/a	Involved in the study
☐	☒ Antibodies
☐	☒ Eukaryotic cell lines
☒	☐ Palaeontology and archaeology
☒	☐ Animals and other organisms
☒	☐ Clinical data
☐	☒ Dual use research of concern
☒	☐ Plants

Methods

n/a	Involved in the study
☒	☐ ChIP-seq
☒	☐ Flow cytometry
☒	☐ MRI-based neuroimaging

Antibodies

Antibodies used	Phycoerythrin (PE)-conjugated goat anti-human antibodies from Southern Biotech: IgG (9040-09), IgG1 (9052-09), IgG2 (9070-09), IgG3 (9210-09), IgG4 (9200-09), IgA1 (9130-09), IgA2 (9140-09), and IgM (9020-09). Anti-human CD66b antibody from BioLegend (305112).
Validation	All antibodies used were validated by the manufacturers. RRID numbers are available for all antibody reagents as follows: AB_2796601 (IgG), AB_2796621 (IgG1), AB_2796639 (IgG2), AB_2796701 (IgG3), AB_2796693 (IgG4), AB_2796656 (IgA1), AB_2796664 (IgA2), AB_2796577 (IgM), AB_2563294 (CD66b).

Eukaryotic cell lines

Policy information about cell lines and Sex and Gender in Research

Cell line source(s)	THP-1 cells (human acute monocytic leukemia cell line, American Type Culture Collection);
Authentication	Cell lines were not authenticated after purchase.
Mycoplasma contamination	Cell lines were not tested for mycoplasma contamination.
Commonly misidentified lines (See ICLAC register)	No commonly misidentified cell lines were used in this study.
